# Can preoperative neutrophil lymphocyte ratio predict malignancy in patients undergoing partial nephrectomy because of renal mass?

**DOI:** 10.1590/S1677-5538.IBJU.2017.0225

**Published:** 2018

**Authors:** Sacit Nuri Gorgel, Kutan Ozer, Osman Kose, Ahmet Selçuk Dindar

**Affiliations:** 1 Department of Urology, Izmir Katip Celebi University, Ataturk Training and Research Hospital, Izmir, Turkey

**Keywords:** Carcinoma, Renal Cell, Neutrophils, Lymphocytes

## Abstract

**Purpose:**

To evaluate the importance of preoperative neutrophil lymphocyte ratio (NLR) predicting malignancy in patients who undergo partial nephrectomy due to renal mass.

**Materials and Methods:**

Seventy nine patients who underwent open partial nephrectomy for renal masses were included in this retrospective study. In preoperative routine blood tests, renal ultrasonography and contrast-enhanced computed tomography were performed for all patients. Preoperative neutrophil lymphocyte ratio were compared in patients with clear cell renal cell carcinoma (Group1, 65 patients) and benign lesions (Group 2, 14 patients). The predictive ability of NLR was analyzed by ROC curves and Youden Index method was used to identify the cut-off value for NLR.

**Results:**

The mean age of patients was 59.8±11.7 years in Group1 and 57.4±12.6 years in Group 2 (p=0.493). The mean tumor size was 3.8±1.2 cm in Group 1 and 3.3±1.0 cm in Group 2 (p=0.07). The median NLR was 2.48 (1.04) in Group 1 and 1.63 (0.96) in Group 2 (p<0.001). The area under a ROC curve was 0.799 (p<0.001).

**Conclusions:**

Preoperative neutrophil lymphocyte ratio may predict renal masses that can not be distinguished radiologically. Our results must be confirmed by large and properly designed prospective, randomized trials.

## INTRODUCTION

Renal cell carcinoma (RCC) represents 2-3% of all cancers (1), with the highest incidence in Western countries. Over the last two decades until recently, the incidence of RCC increased by about 2% both worldwide and in Europe, although a continuing decrease has been observed in Denmark and Sweden (2). In 2012, there were approximately 84.400 new cases of RCC and 34.700 kidney cancer-related deaths in the European Union (3). In Europe, overall mortality rates for RCC increased up to the early 1990s, and stabilized or declined thereafter (4). Due to increased detection of tumors by ultrasound (US) and computed tomography (CT), the number of incidentally diagnosed RCCs has in-creased. These tumors are usually smaller and of lower stage (5-7).

The relation between inflammation and tumor development and progression has been recognized in recent decades (8, 9). As a typical representative of inflammatory reactions, C-reactive protein (CRP) has been reported to be significantly associated with the prognosis of several cancers (10-14). Other systematic inflammation markers have been validated as predictive in various types of cancer (15-17).

The neutrophil to lymphocyte ratio (NLR) is also a widely used inflammatory marker that is defined as the absolute neutrophil count divided by the absolute lymphocyte count, and can be easily determined from complete blood cell parameters (18).

Unfortunately, radiological methods are still not sufficient for predicting malignancy. This can lead to unnecessary surgery in patients with benign renal masses. In this study, we investigated the importance of preoperative neutrophil lymphocyte ratio predicting malignancy in undistinguished radiological renal masses. This is the first study in the literature.

## MATERIALS AND METHODS

Seventy nine patients who underwent open partial nephrectomy for renal masses were included in the study between 2006 and 2015. Patient data were analyzed retrospectively. In preoperative routine blood tests, renal ultrasonography and contrast-enhanced computed tomography were performed in all patients. NLR was compared in patients with clear cell RCC (Group 1, 65 patients) and benign lesions (Group 2, 14 patients). The predictive ability of NLR was analyzed by ROC curves and Youden Index method was used to identify the cut-off value for NLR.

### Statistical Method

Normality distribution was investigated for all numeric variables. Categorical variables were described by frequencies and percentages and numeric variables were described by means and standard deviations or medians and interquartile ranges. The relationship between two categorical variables was tested by Chi-square test. Two independent means was compared by Student t test and two independent medians were compared by Mann Whitney U test. The predictive ability of NLR was analyzed by ROC curves and Youden Index method was used to identify the cut-off value for NLR. A p value less than 05 was accepted as statistically significant.

## RESULTS

Mean age of patients was 59.8±11.7 years in Group 1 and 57.4±12.6 years in Group 2 (p=0.493). Mean tumor size was 3.8±1.2cm in Group 1 and 3.3±1.0cm in Group 2 (p=0.07). The median NLR was 2.48 (1.04) in group 1 and 1.63 (0.96) in group 2 (p<0.001). Both groups were similar in terms of sex and tumor side (Table-1) 14 patients had benign lesions. Eight patients had oncocytoma, 3 patients had calcified cyst, 2 patients had osseous metaplasia and 1 patient had angiomyolipoma.

**Table 1:** Descriptive characteristics of the patients.

		Group 1 (n=65)	Group 2 (n=14)	p
Age(Mean±SD)		59.8±11.7	57.4±12.6	0.493
**Sex(n, %)**				
	Male	35 (53.8)	6 (42.9)	0.455
	Female	30 (46.2)	8 (57.1)	
**Side(n, %)**				
	Right	35 (53.8)	7 (50)	0.794
	Left	30 (46.2)	7 (50)	
Size (cm)(Mean±SD)		3.8±1.2	3.3±1.0	0.077
NLR(Median, IQR)		2.48 (1.04)	1.63 (0.96)	<0.001

**RCC** = Renal cell carcinoma; **SD** = Standard deviation; **IQR** = Interquartile range

Forty three (66.2%) of 65 patients had pT1a tumor and 22 (33.8%) of 65 patients had pT1b tumor 15 patients had grade 1 tumor, 46 patients had grade 2 tumor and 4 patients had grade 3 tumor. Optimal cut-off value of NLR was 1.725, with sensitivity of 93.8% and specificity 64.3% (Table-2).

**Table 2:** Cut-off values of NLR for malignancy.

	Cut-off values	Sensitivity %	Specificity %
Optimal	1.725	93.8	64.3
Max Sensitivity	1.275	100	7.1
Max Specificity	3.035	29.2	100

**NLR** = neutrophil lymphocyte ratio

**Figure 1:**
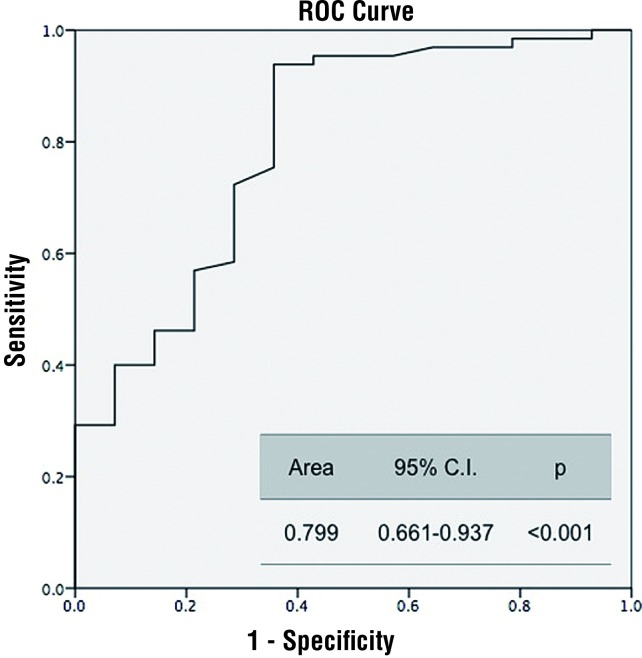
NLR predictive probability of the malignancy. **NLR** = neutrophil lymphocyte ratio

**Figure 2:**
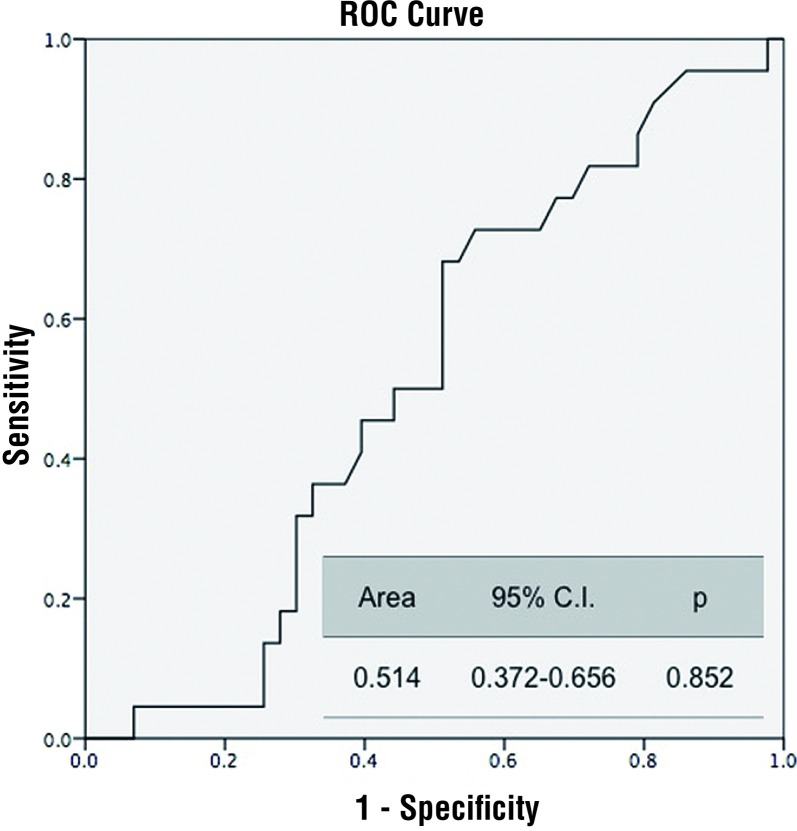
NLR predictive probability of tumor stage. **NLR** = neutrophil lymphocyte ratio

**Figure 3:**
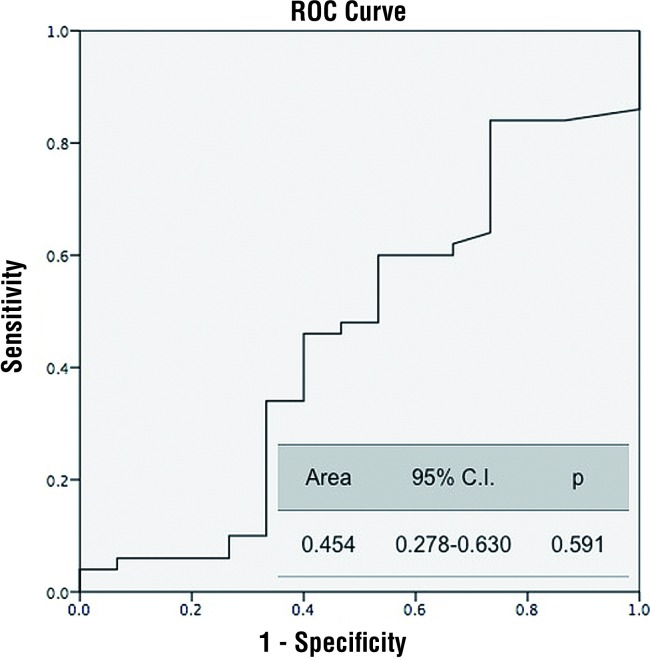
NLR predictive probability of the tumor grade. **NLR** = neutrophil lymphocyte ratio

The area under the ROC curve was 0.799 (p<0.001) (Figure-1). Predictive probability of NLR for tumor stage and grade were statistically insignificant (p=0.852) (Figures 2 and 3).

## DISCUSSION

Increasing evidences support the involvement of systemic inflammation in cancer development and progression (9). It has been shown that, among the leukocytes in circulation, neutrophils increase and lymphocytes decrease as a systemic inflammatory response develops against the tumor. NLR has been used as an indicator of systemic inflammatory response (18).

An elevated NLR reflects both a decreased lymphocyte mediated antitumor immune response and a increased neutrophil dependent inflammatory reaction. Both of these factors may contribute to aggressive tumor biology, cancer progression, and poor prognosis (19, 20).

Despite recent progress in the identification of genetic, epigenetic and common molecular alterations in RCC (21), routine diagnostic and prognostic assessment of RCC currently relies on pathological tissue examination and traditional clinicopathological prognostic variables (22). The complexity of these molecular changes, as well as high costs of analyses, time-consuming preparation required and lack of evidence demonstrating how these newly discovered molecular markers influence diagnostic or therapeutic decisions, have rendered none of the markers available for routine testing.

Recently, several serum biomarkers and hematological indices representative of inflammatory response, notably C reactive protein (CRP), fibrinogen, lymphocyte-monocyte ratio, neutrophil-lymphocyte ratio (NLR) and platelet-lymphocyte ratio, have been demonstrated to be closely related to poor prognosis of patients with RCC (23-25). Therefore, NLR, defined as neutrophil counts divided by lymphocyte counts, is particularly noteworthy. Emerging evidences demonstrated that NLR showed its prognostic value in patients with colorectal cancer (26) and hepatocellular carcinoma (27). Patients with RCC with elevated levels of pretreatment NLR may be more likely to present a poorer clinical outcome (28).

There are other laboratory markers of systemic inflammation reaction besides NLR, such as CRP (29) and modified Glasgow prognostic score (30, 31), with a prognostic role in patients with RCC. Also, gene polymorphisms (32) and biological markers (33, 34) are suggested to be predictors of prognosis in patients with RCC. Although NLR is easy to measure, conditions such as active infection, inflammatory diseases, smoking behavior or stress at the time of blood collection may affect it (35).

In previous studies, it was demonstrated a relationship between poor prognosis and NLR. Unlike previous studies, we investigated the role of NLR in the malign-benign distinction. In the present study, we found that preoperative neutrophil lymphocyte ratio may predict renal masses that cannot be distinguished radiologically.

This study has several limitations. First, the study was retrospective. Second, NLR could be affected by different conditions, especially undetected diseases such as chronic infection, chronic disease, and autoimmune disorders, such as rheumatic disease. Third, the number of patients was especially low in the benign group. Our results should be confirmed by prospective randomized studies with large population patients. If confirmed, preoperative NLR will be an important tool to prevent unnecessary surgeries.

## CONCLUSIONS

Preoperative neutrophil lymphocyte ratio might predict renal masses that cannot be distinguished radiologically. Our results must be confirmed by large and properly designed prospective, randomized trials.
